# SUN Family Proteins Sun4p, Uth1p and Sim1p Are Secreted from *Saccharomyces cerevisiae* and Produced Dependently on Oxygen Level

**DOI:** 10.1371/journal.pone.0073882

**Published:** 2013-09-11

**Authors:** Evgeny Kuznetsov, Helena Kučerová, Libuše Váchová, Zdena Palková

**Affiliations:** 1 Department of Genetics and Microbiology, Faculty of Science, Charles University in Prague, Prague, Czech Republic; 2 Institute of Microbiology of the ASCR, v.v.i., Prague, Czech Republic; Institute of Biology Valrose, France

## Abstract

The SUN family is comprised of proteins that are conserved among various yeasts and fungi, but that are absent in mammals and plants. Although the function(s) of these proteins are mostly unknown, they have been linked to various, often unrelated cellular processes such as those connected to mitochondrial and cell wall functions. Here we show that three of the four *Saccharomyces cerevisiae* SUN family proteins, Uth1p, Sim1p and Sun4p, are efficiently secreted out of the cells in different growth phases and their production is affected by the level of oxygen. The Uth1p, Sim1p, Sun4p and Nca3p are mostly synthesized during the growth phase of both yeast liquid cultures and colonies. Culture transition to slow-growing or stationary phases is linked with a decreased cellular concentration of Sim1p and Sun4p and with their efficient release from the cells. In contrast, Uth1p is released mainly from growing cells. The synthesis of Uth1p and Sim1p, but not of Sun4p, is repressed by anoxia. All four proteins confer cell sensitivity to zymolyase. In addition, Uth1p affects cell sensitivity to compounds influencing cell wall composition and integrity (such as Calcofluor white and Congo red) differently when growing on fermentative versus respiratory carbon sources. In contrast, Uth1p is essential for cell resistance to boric acids irrespective of carbon source. In summary, our novel findings support the hypothesis that SUN family proteins are involved in the remodeling of the yeast cell wall during the various phases of yeast culture development and under various environmental conditions. The finding that Uth1p is involved in cell sensitivity to boric acid, i.e. to a compound that is commonly used as an important antifungal in mycoses, opens up new possibilities of investigating the mechanisms of boric acid’s action.

## Introduction

The SUN family of 4 genes, (*SIM1*, *UTH1*, *NCA3* and *SUN4*) coding for homologous proteins similar to cell wall glucanases, is related to a variety of cellular processes. These proteins share a C-terminal amino acid domain (about 258 amino acid long) that is highly homologous among the members of the group (75–85% amino acid identity). This domain contains a putative Fe-binding domain with 4 cysteins (Cys-X5-Cys-X3-Cys-X24-Cys motif) [1]. The most studied member of the group *UTH1* was first described as a yeast-ageing gene, the deletion of which confers increased resistance to various stresses, including high temperature and oxidative stress, prolonged replicative life-span and increased mutant cell longevity [2]–[5]. Surprisingly, Uth1p was described to have a dual localization in the cell wall and in the outer mitochondrial membrane [6]. Uth1p was suggested to be important for mitochondrial autophagy (mitophagy) [7] and the presence of this protein was required for a proapoptotic effect of the mammalian BAX protein when expressed in yeast [4]. These findings, together with the observation that *uth1*Δ cells reduce the level of some mitochondrial proteins, such as cytochromes and citrate synthase, indicate that Uth1p may be a regulator of mitochondrial function. On the other hand, other findings suggest that Uth1p, like some other members of SUN family, may affect the function of the yeast cell wall. *uth1*Δ cells were described to have a cell wall that differs in its β-D-glucan and chitin composition from wild-type cells and that is more resistant to zymolyase treatment [8].

Little is known of the function of the three other members of SUN family, Nca3p, Sim1p and Sun4p. The function of Nca3p may be also related to mitochondria, as this protein was identified as a multicopy suppressor of a deficiency in the mitochondrial synthesis of some subunits of the ATP synthase [9]. Sim1p may be somehow involved in the regulation of cyclin-dependent kinase activity, since the *sim1*Δ strain with additional deletions of cyclines Clb1p and Clb4p exhibited an altered replication [10]. Sim1p when overproduced from multicopy plasmid, functioned also as a high copy extracellular suppressor of mutations in *PAG1* and *CBK1* genes involved in cellular morphogenesis [11]. Like Uth1p, Sun4p/Scw3p also exhibits a dual localization, being identified in mitochondria and in the cell wall, and this protein is supposed to be involved in cell septation [12], [13]. All SUN family proteins are homologous to the β-glucosidase of *Candida wickerhamii,* but no proof of their β-glucosidase activity has yet been found [14].

The SUN family protein Sun41p from *C. albicans* plays a role in cell attachment to a substrate and in biofilm formation. The *sun41*Δ strain forms aberrant hyphae and has decreased virulence [15]. A later study revealed that Sun41p is involved in cell separation and hyphal differentiation in *C. albicans*, and it exhibits synthetic lethality with Sun42p, another SUN family member of *C. albicans*. It has been therefore proposed that the SUN proteins of *Candida* sp. could be involved in cell wall remodeling linked to the maintenance of cell integrity during cell division [16].

Despite these mostly fragmentary data that relate the function of SUN proteins to either the cell wall or to mitochondria, the actual function of these proteins is currently unknown. Here we show that three members of the *S. cerevisiae* SUN family, Uth1p, Sim1p and Sun4p, are effectively released from cells growing either in liquid or on solid media. In addition, the production of Uth1p and Sim1p is controlled by oxygen concentration. The absence of each of the SUN proteins affects cell resistance to zymolyase and some other compounds affecting the cell wall. In addition, the absence of Uth1p markedly increases cell sensitivity to boric acid, a fungistatic agent widely used in the treatment of vaginal yeast infections. This effect is reversed by Uth1p overexpression. Our findings support the hypothesis of the function of SUN proteins being related to the function of the cell wall and indicate that Uth1p in particular could be involved in resistance to boric acid and thus could be a potential target in mycose treatment.

## Results

### Three of Four SUN Proteins are Secreted from Cells Grown in Liquid Cultures

SUN family genes code for proteins that are homologous, but involved in various unrelated processes and could act in different cellular compartments. Table 1 summarizes predicted features of these proteins, such as their mutual homology, Mw, presence of signaling secretion sequence and of glycosylation sites. Each of the SUN proteins contains potential signaling secretion sequence on their N-terminus and could be highly O-mannosylated. In addition, Sun4p and Nca3p could be N-glycosylated. These features suggest that all SUN proteins could be routed through the secretory pathway.

**Table 1:** Predicted properties of SUN family proteins.

Properties of SUN proteins	Uth1p	Sun4p	Sim1p	Nca3p	
Predicted Mw (kDal)	36.955	43.442	48.19	35.412	http://www.yeastgenome.org/
Putative signal peptide for secretion	1–17 AA	1–22 AA	1–19AA	1–18AA	http://www.yeastgenome.org/
N-glycosylation sites	0	1 (395 AA)	0	1 (117 AA)	http://www.oppf.ox.ac.uk/opal/
O-mannosylation sites	28	49	78	9	http://www.oppf.ox.ac.uk/opal/
Kex2 cleavage sites (LysArg)	1(32–33 AA)	1(44–45 AA)	1(34–35 AA)	1(76–77 AA)	
**Percent identity matrix of SUN proteins**	**Uth1p**	**Sun4p**	**Sim1p**	**Nca3p**	http://www.ebi.ac.uk/Tools/msa/clustalo/
**Uth1p**	100	56.42	59.15	66.27	
**Sun4p**	56.42	100	72.20	54.93	
**Sim1p**	59.15	72.20	100	58.41	
**Nca3p**	66.27	54.93	58.41	100	

AA…. amino acid.

To monitor the production of SUN proteins, we prepared *S. cerevisiae* BY4742 strains containing *SUN4*, *UTH1*, *SIM1* and *NCA3* genes fused with a HA tag directly in the genome (Table 2). Genomic SUN-HA fusions guarantee the stability of the constructs as well as the natural regulation of SUN gene expression and of the amounts of proteins produced. The HA-tag did not change the functionality of the particular protein, as it did not affect the properties of the strains compared to the parental strain (data not shown).

**Table 2:** Strains.

Strain	Genotype	source
BY4742 (wt)	*MATα*, *his3*Δ*1*, *leu2*Δ*0*, *lys2*Δ*0*, *ura3*Δ*0*	EUROSCARF
BY-Uth1p-HA	*MATα*, *his3*Δ*1*, *leu2*Δ*0*, *lys2*Δ*0*, *ura3*Δ*0, UTH1-6HA-HIS3MX6*	this study
BY-Nca3p-HA	*MATα*, *his3*Δ*1*, *leu2*Δ*0*, *lys2*Δ*0*, *ura3*Δ*0, NCA3-6HA-HIS3MX6*	this study
BY-Sun4p-HA	*MATα*, *his3*Δ*1*, *leu2*Δ*0*, *lys2*Δ*0*, *ura3*Δ*0, SUN4-6HA-HIS3MX6*	this study
BY-Sim1p-HA	*MATα*, *his3*Δ*1*, *leu2*Δ*0*, *lys2*Δ*0*, *ura3*Δ*0, SIM1-6HA-HIS3MX6*	this study
BY-p_TEF_-*UTH1*	*MATα*, *his3*Δ*1*, *leu2*Δ*0*, *lys2*Δ*0*, *ura3*Δ*0 p_TEF_-UTH1-kanMX18*	this study
BY-*uth1*Δ	*MATα*, *his3*Δ*1*, *leu2*Δ*0*, *lys2*Δ*0*, *ura3*Δ*0, uth1*Δ*-kanMX4*	EUROSCARF
BY-*nca3*Δ	*MATα*, *his3*Δ*1*, *leu2*Δ*0*, *lys2*Δ*0*, *ura3*Δ*0, nca3*Δ*-kanMX4*	EUROSCARF
BY-*sun4*Δ	*MATα*, *his3*Δ*1*, *leu2*Δ*0*, *lys2*Δ*0*, *ura3*Δ*0, sun4*Δ*-kanMX4*	EUROSCARF
BY-*sim1*Δ	*MATα*, *his3*Δ*1*, *leu2*Δ*0*, *lys2*Δ*0*, *ura3*Δ*0, sim1*Δ*-kanMX4*	EUROSCARF

**Figure 1:**
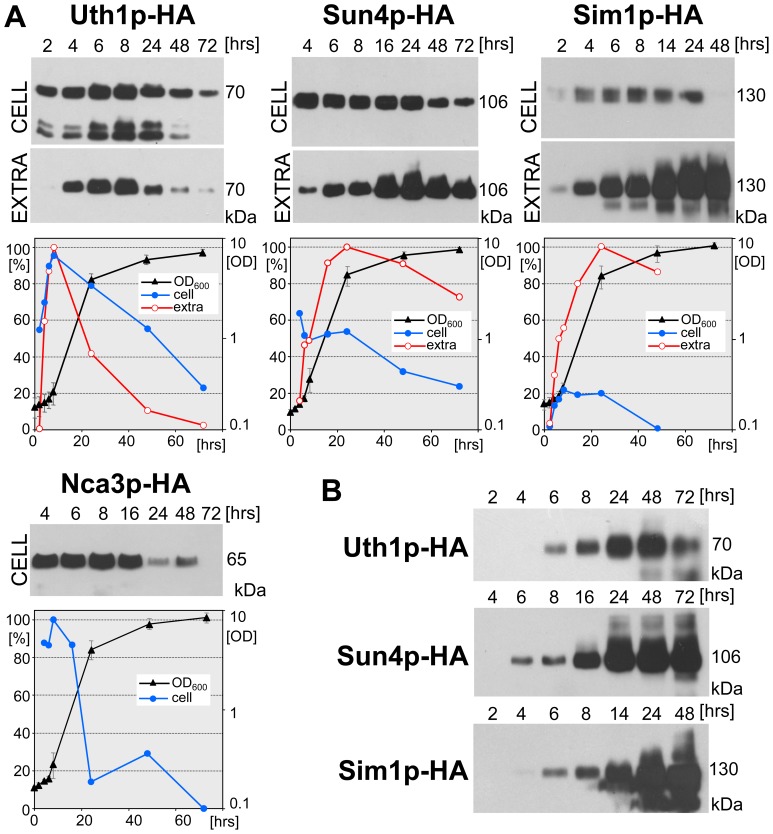
Cellular/extracellular level of individual SUN proteins during growth of liquid cell cultures in GM medium. A , Protein amounts in cell lysates (CELL) and in extracellular extracts (EXTRA), respectively, prepared from liquid cell cultures of BY-Uth1p-HA, BY-Sun4p-HA, BY-Sim1p-HA strains and the protein amounts in cell lysates from a liquid cell culture of BY-Nca3p-HA strain. The standardized amounts of lysate-proteins or the corresponding amounts of extracellular extracts were loaded onto the gel (loading controls, see Figure S3 A). A representative Western blot of 3 to 4 independent biological replicates is shown. The highest amount of the particular protein in each immunoblot quantified by densitometry was set as 100%. Growth curves of cultures of individual strains are shown (right axis). **B**, extracellular accumulation of Sun4p-HA, Uth1p-HA and Simp1p-HA proteins. Proteins precipitated from a constant volume of the medium were loaded onto the gel. A representative Western blot of 2 to 4 independent biological replicates is shown. The value of Mw at the right side of immunoblots represents Mw of particular SUN protein linked to the HA tag. S.D. values were calculated from 3–5 independent biological replicates.

Three of the four SUN proteins have been related to mitochondria either functionally or by their cellular localization [1], [4], [7], [17]–[19]. Therefore, we monitored the level and timing of production of individual SUN proteins in respiratory GM medium with glycerol as a non-fermentative carbon source. We harvested cells for lysate preparation and protein analysis on Western blots at particular time-points throughout the growth of the shaken liquid cultures of each of the four strains. In parallel, we took aliquots of cell-free cultivation medium for an analysis of extracellular proteins. To be able to quantify the amount of SUN proteins released by cells to the cultivation medium and compare it with the amount of cellular SUN proteins, we performed an SDS-PAGE on the amount of extracellular proteins precipitated from a culture volume equivalent to the amount of biomass used for the preparation of the lysate (see Materials and Methods) (Figure 1A). In addition, we followed the time course of accumulation of SUN proteins in the cultivation medium as the amount of them present in constant aliquots taken at each time-point, as shown in Figure 1B.

Figure 1A shows that three of the four SUN proteins, Uth1p-HA, Sun4p-HA and Sim1p-HA are released to the cultivation medium with high efficiency. On the other hand, no Nca3p-HA was detected extracellularly during the cultivation (data not shown). Individual SUN proteins differed in the ratio of their cellular/extracellular protein concentration as well as in the timing of their production in the various growth phases. The cellular levels of all SUN proteins started to increase early during the cultivation, with Sim1p-HA being the latest. The cellular level of Uth1p-HA (Uth1p-HA^cell^) gradually increased and reached its maximal values during the early logarithmic growth phase (after 6–10 hrs of cultivation). Later, at the end of the logarithmic growth phase, Uth1p-HA^cell^ started to decrease, however, some protein was still present even at 72 h. The extracellular Uth1p-HA level (Uth1p-HA^ex^), i.e. the amount of Uth1p-HA produced by a biomass unit that is released from the cells to the extracellular medium, approximately matched the cellular level profile. Cellular Sun4p-HA (Sun4p-HA^cell^) and Sim1p-HA (Sim1p-HA^cell^) remained at about the same high level from approximately the 4^th^ to 24^th^ h of cultivation, i.e. to the end of the exponential growth phase. After this point, Sun4p-HA^cell^ dropped to about half of its exponential-phase level; Sim1p-HA^cell^, however, almost disappeared from the cells. The amount of both Sun4p-HA^ex^ and Sim1p-HA^ex^ released by the unit of biomass increased, this increase beginning at the end of the exponential growth phase. Cellular Nca3p-HA (Nca3p-HA^cell^) was kept at a constant level, similarly to Sun4p-HA^cell^ and Sim1p-HA^cell^; its level began to decrease from the 24^th^ h onwards. The finding of different levels of extracellular SUN proteins released from the cells of post-exponential and stationary cultures is consistent with the observation that the extracellular accumulation (i.e. amount of protein in a constant volume of the medium) of Sun4p-HA^ex^ and Simp1p-HA^ex^ in the medium was still high even after 3 days of cultivation, while the absolute level of Uth1p-HA^ex^ in the medium decreased (Figure 1B).

**Figure 2:**
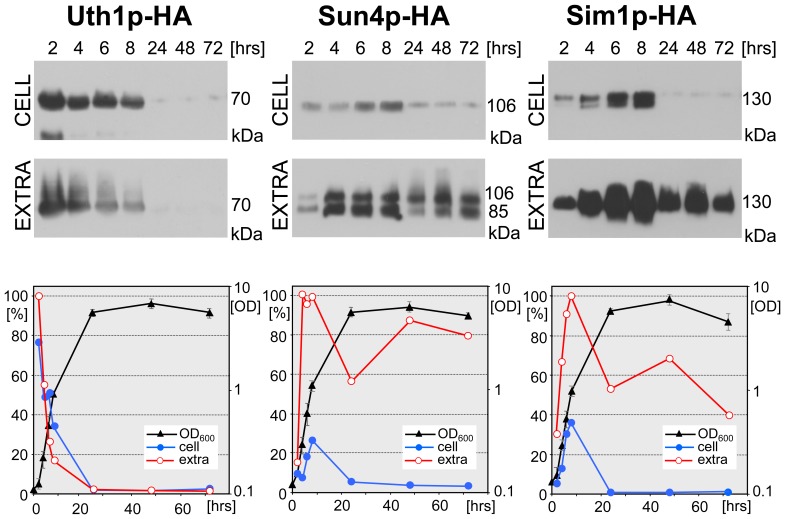
Cellular/extracellular level of SUN proteins during growth of liquid cell cultures in glucose YED medium. Protein amounts in cell lysates (CELL) and in extracellular extracts (EXTRA), respectively, prepared from liquid cell cultures of BY-Uth1p-HA, BY-Sun4p-HA and BY-Sim1p-HA strains. The standardized amounts of lysate-proteins or the corresponding amounts of extracellular extracts were loaded onto the gel (loading controls see Figure S3 B). A representative Western blot of the 2 independent biological replicates is shown. The highest amount of the particular protein in each immunoblot quantified by densitometry was set as 100%. Growth curves of individual strains are shown (right axis). The value of Mw at the right side of immunoblots represents Mw of particular SUN protein linked to the HA tag.

To test whether the SUN protein production and secretion differs in respiratory and fermentative media, we analyzed the level of cellular and extracellular SUN proteins in a liquid cell culture grown in YED medium. As shown in Figure 2, Uth1p-HA, Sun4p-HA and Sim1p-HA are produced in YED medium during the exponential phase of population growth and are released out of the cells with profile similar to that observed in GM medium (Figure 1). Thus, in spite of differences related to different grow-rate of yeast in respiratory versus fermentative medium, general profiles of production and release of SUN proteins are similar in both media.

### Uth1p, Sun4p and Sim1p are Present in Extracellular Space of Developing Colonies

Time-line analyses of SUN protein production and localization (especially the ability to release the proteins from the cells) in yeast shaken liquid cultures revealed significant differences between the individual SUN proteins. We therefore asked the question of when these proteins are produced and where they localize in multicellular yeast colonies that exhibit typical linear growth on solid nutrient agar [20], [21]. We therefore harvested colonies at various developmental time-points and prepared the two protein samples; extracellular proteins washed out from the cells and the cell lysate. Both fractions were analyzed for the presence of SUN proteins using Western blots. As with the samples from liquid cultures, the amount of extracellular proteins from colonies that were loaded into the SDS-PAGE was produced by approximately the same amount of cell biomass that was used to prepare the cell lysate loaded for the detection of cellular SUN proteins.

**Figure 3:**
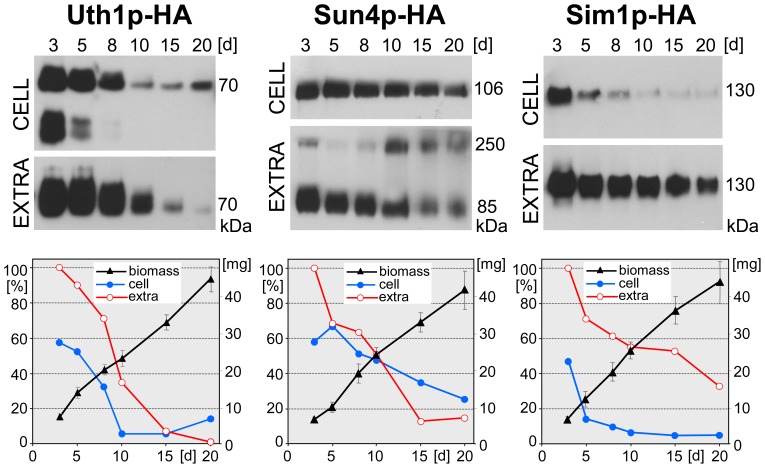
Cellular/extracellular level of individual SUN proteins during development of colonies growing on GMA plates. Protein amounts in cell lysates (CELL) and in extracellular extracts (EXTRA), respectively, prepared from colonies of BY-Uth1p-HA, BY-Sun4p-HA and BY-Sim1p-HA strains. The standardized amounts of lysate-proteins were loaded onto the gel (loading controls, see Figure S3 C) and corresponding amounts of extracellular extracts. The representative Western blot of 3–4 independent biological replicates is shown. The highest amount of the particular protein in each immunoblot quantified by densitometry was set as 100%. Growth curves of colonies formed by individual strains are shown as wet weight per one colony (right axis). The value of Mw at the right side of immunoblots represents Mw of particular SUN protein linked to the HA tag. S.D. values were calculated from 3–4 independent biological replicates.

While the amount of Nca3p-HA within developing colonies was below the detection limit of the method used, we detected a significant level of both cellular and extracellularly released Uth1p-HA, Sun4p-HA and Sim1p-HA (Figure 3). As in liquid cultures, the profile of the presence of these proteins within colonies differed significantly. Uth1p-HA was present in high levels in relatively young colonies (3-8-days-old) and was released into the extracellular space from the beginning. From day 10 of colony development, Uth1p-HA^cell^ significantly decreased and the protein also disappeared from the extracellular space of the colony from about the 15^th^ day. Sun4p-HA was also found in colonies from the early phases of their growth, but in contrast to both Uth1p-HA and Sim1p-HA, its cellular level was constant until the late developmental phases (20-days-old colonies). Sun4p-HA^ex^ followed approximately the same profile, with a significant decrease in 15- and 20-days-old colonies. Only Sim1p-HA^ex^ persisted at a constant relatively high concentration until the end of the monitored period of colony development (20-days-old colonies). On the other hand, Sim1p-HA^cell^ dropped quickly, being only high in young colonies (3-days-old) and becoming almost undetectable from day 8 of colony development. The absence of any of the three SUN proteins did not affect significantly viability of cells within colonies (Figure S1).

### SUN Genes are Regulated Differently according to the Level of Oxygen

**Figure 4:**
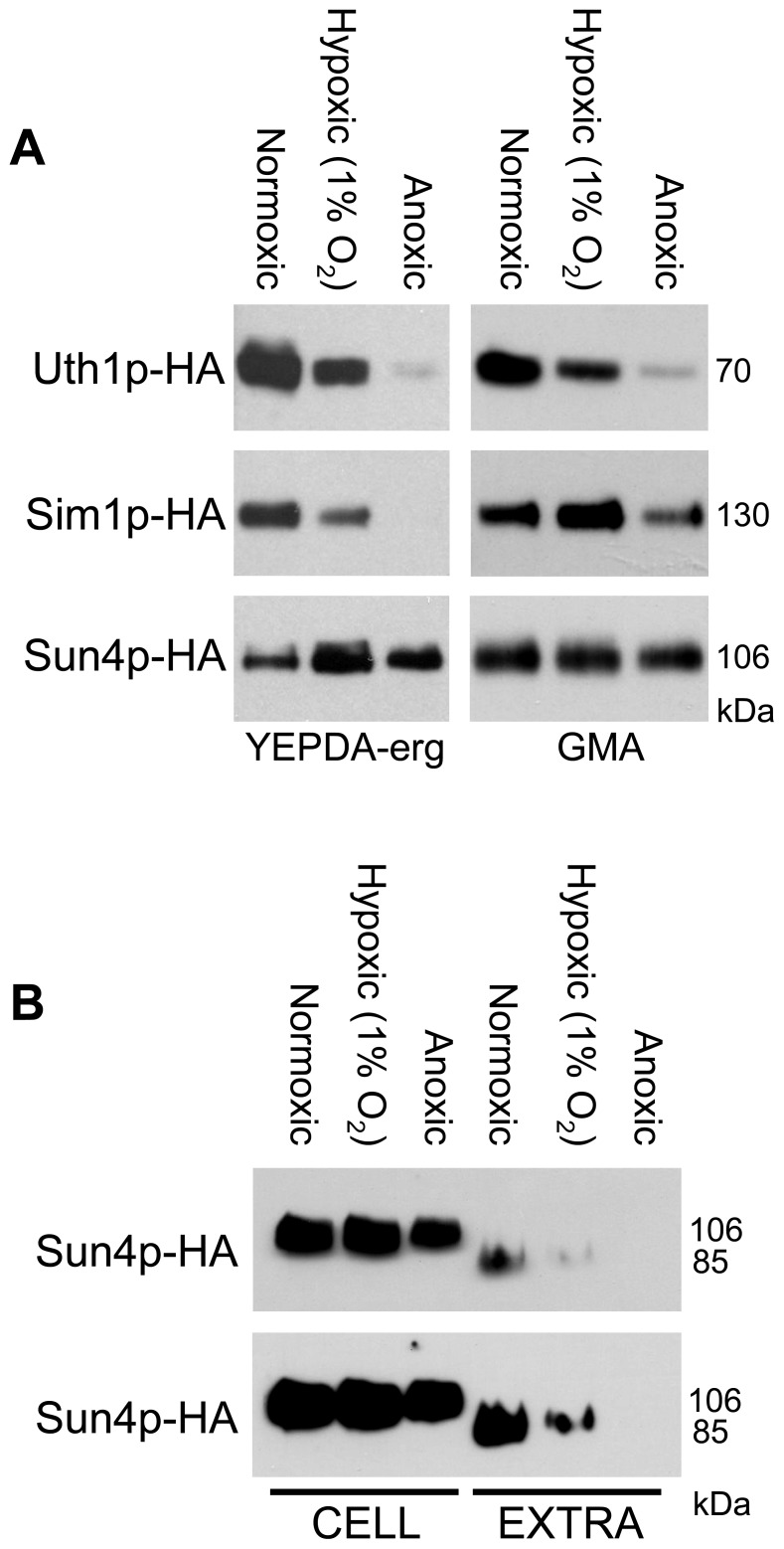
O_2_ concentration affects SUN protein production. **A**, Protein amounts in cell lysates of unwashed colonies of BY-Uth1p-HA, BY-Sun4p-HA and BY-Sim1p-HA strains. Strains were either grown on YEPDA-erg or incubated on GMA under normoxic, hypoxic or anoxic atmosphere. The representative Western blot of the 2 independent biological replicates is shown. **B**, Sun4p-HA amounts in cell lysates (CELL) and in extracellular extracts (EXTRA) from colonies. BY-Sun4p-HA strain was grown on YEPDA-erg under normoxic, hypoxic or anoxic conditions for 3 days. Two film-expositions are shown to better visualize differences in protein concentrations in individual samples. The value of Mw at the right side of immunoblots represents Mw of particular SUN protein linked to the HA tag. The standardized amounts of lysate-proteins or the corresponding amounts of extracellular extracts were loaded onto the gel (loading controls, see Figure S3 D).

The function of some of the SUN proteins was related to the function of mitochondria [1], [4], [7], [17]–[19] and in addition, there are indications that the expression of the *SIM1* gene from *Candida albicans* is induced under hypoxic conditions [22]. We therefore analyzed the production of the three SUN proteins that were detected at relatively high levels within colonies (Uth1p-HA, Sim1p-HA and Sun4p-HA) under various levels of oxygen tension, i.e. normoxic, hypoxic (1% O_2_) and anoxic conditions. Two experimental setups were designed. First, colonies of the strains with HA-tagged versions of the SUN proteins were grown for 3 days in parallel under normoxic and anoxic conditions and in an atmosphere with 1% O_2_ on YEPDA-Erg with glucose as the fermentative carbon source supplemented with ergosterol, a compound essential for yeast growth under anoxic conditions [23]. Figure 4A shows that the total level (comprised of both cellular and extracellular protein) of both Uth1p-HA and Sim1p-HA decreased with decreased oxygen concentration, being negligible under anoxic conditions. On the other hand, the total level of Sun4p was relatively stable, or even slightly higher under conditions of decreased oxygen tension. However, extracellular Sun4p-HA was not detected in anoxic conditions and the level of Sun4p-HA^ex^ in hypoxia was significantly lowered when compared to normoxic conditions (Figure 4B). This finding indicates that either extracellular secretion of this protein is decreased or Sun4p-HA^ex^ is more efficiently degraded in anoxia/hypoxia.

In a parallel experiment, colonies were grown for 3 days on respiratory GMA plates under normoxic conditions and then the colonies were transferred to the anoxomat device and incubated for an additional 2 days under low oxygen, i.e. under conditions where the cells cannot divide and grow. Figure 4A shows that the level of Uth1p and Sim1p also partially dropped under these non-growing conditions, while the level of Sun4p remained stable.

### SUN-protein-deficient Yeast Strains Differ in Properties of their Cell Walls

**Figure 5:**
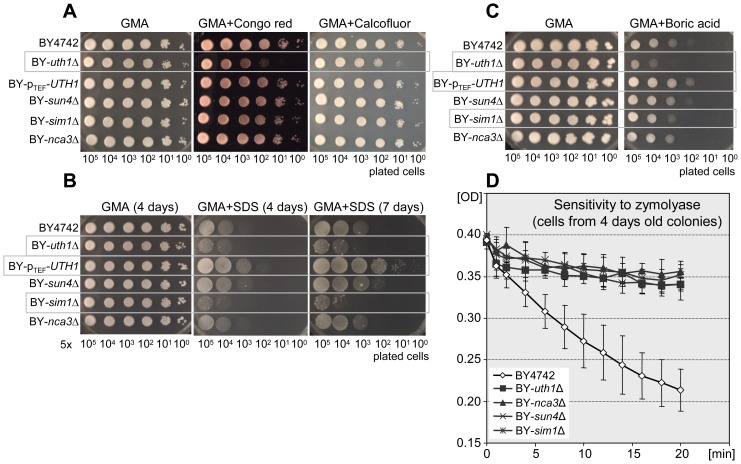
Strains deficient in SUN proteins have different sensitivity to zymolyase and various drugs when grown on respiratory GMA agar. A–C, drop assays of cells of BY4742 (wt), BY-*uth1*Δ, BY-p_TEF_-*UTH1*, BY-*sun4*Δ, BY-*sim1*Δ and BY-*nca3*Δ strains on GMA plates supplemented with various drugs. Representative experiments of at least three biological replicates are presented. Significant drug effects on particular strains are marked by grey boxes; the quantification of the drug effect is shown in Figure S4A. **A**, effect of Congo red (800 µg/ml) or Calcofluor white (1 mg/ml); cells were grown at 37°C for 4 days. **B**, effect of SDS (0.012%), cells were grown at 28°C for 4 or 7 days as indicated. **C**, effect of boric acid (0.4%), cells were grown at 28°C for 7 days. **D**, cell resistance to zymolyase (0.2 U/µl) presented as decrease in density of cell suspension. S.D. values were calculated from four independent biological replicates for the mutant strains and 10 replicates for the wt; the significance of the difference between BY4742 and the other four strains was determined using two-way ANOVA with p≤0.0001.

Previous data also suggested that Uth1p and Sun4p proteins localize to the cell wall and may affect its properties [6], [8]. We therefore analyzed the sensitivity of the strains individually deleted in each of the SUN genes to a spectrum of compounds that are known to be cytostatic for yeast, affecting their cell wall, plasma membrane or other cellular processes (Figure 5).

Figure 5D shows that a deletion of any of the SUN genes significantly increases yeast cell resistance to zymolyase treatment. The resistance was determined using cells from 4-days-old colonies growing on GMA. On the other hand, only the BY-*uth1*Δ strain was significantly more sensitive to Congo red or Calcofluor white (CFW) dyes when present in cultivation GMA agar (Figures 5A and S4A). The effect of these compounds that affect the cell wall was more prominent at a higher temperature (37°C) than at 28°C (not shown). This effect was suppressed in the BY-p_TEF_-*UTH1* strain containing the *UTH1* gene under the control of the constitutive *TEF* promoter. BY-*uth1*Δ was also more sensitive to a low concentration of SDS (0.012%) than the wild type strain BY4742, while *UTH1* gene overexpression increased the BY-p_TEF_-*UTH1* strain resistance tenfold compared to the wild-type strain (Figures 5B and S4A). In addition to BY-*uth1*Δ, the BY-*sim1*Δ strain was also significantly more sensitive to SDS than the wild-type strain. BY-*sun4*Δ and BY-*nca3*Δ strains exhibited the same resistance to SDS as the wild-type strain.

Boric acid, being an important antimicrobial, is often used as an antifungal compound in the treatment of vulvovaginal mycoses. Although current knowledge on the mechanisms of boric acid’s action is still fragmentary, indications exist that among other things, this compound affects yeast morphogenesis. Figures 5C and S4A show that the BY-*uth1*Δ strain is about 500-fold more sensitive to 0.012% boric acid and the BY-p_TEF_-*UTH1* strain is about 10 times more resistant to boric acid treatment than the wild-type strain. Of the other SUN knockout strains, only the BY-*sim1*Δ strain was slightly more sensitive to boric acid; BY-*sun4*Δ and BY-*nca3*Δ did not exhibit any differences.

### Sensitivities of SUN-protein-deficient Strains to Toxic Compounds Differ when Cells Grow on Different Carbon Sources, Irrespective of SUN Protein Cellular Concentration

**Figure 6:**
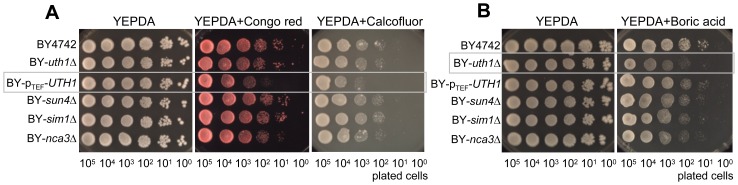
Sensitivity of strains deficient in SUN proteins to toxic compounds on fermentative YEPDA medium. **A, B**, Drop assays of cells of BY4742, BY-*uth1*Δ, BY-p_TEF_-*UTH1*, BY-*sun4*Δ, BY-*sim1*Δ and BY-*nca3*Δ strains on YEPDA plates supplemented with various drugs. Representative experiments of at least three biological replicates are presented. Significant drug effects on particular strains are marked by grey boxes; the quantification of the drug effect is shown in Figure S4 B. **A**, Congo red (800 µg/ml) or Calcofluor white (600 µg/ml), cells were grown at 28°C for 4 days. **B**, boric acid (0.2%), cells were grown at 28°C for 5 days.

**Figure 7:**
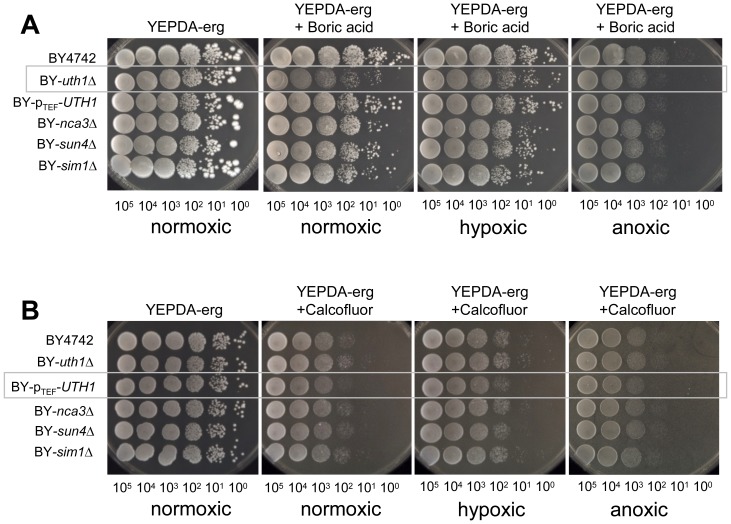
Sensitivity of strains deficient in SUN proteins to toxic compounds on fermentative medium under various levels of oxygen tension. Drop assays of cells of BY4742, BY-*uth1*Δ, BY-p_TEF_-*UTH1*, BY-*sun4*Δ, BY-*sim1*Δ and BY-*nca3*Δ strains on YEPDA-erg plates supplemented with either boric acid or Calcofluor white and grown under normoxic, hypoxic (1% O_2_) or anoxic conditions. Representative experiments of three biological replicates are presented. Significant drug effects on particular strains are marked by grey boxes. **A**, boric acid (0.4%), cells were grown at 28°C for 6 days. **B**, Calcofluor white (2 mg/ml), cells were grown at 28°C for 3 days.

Data obtained on the sensitivity of BY-*uth1*Δ partially contradict the previous findings of [8] that showed an increased resistance of the *uth1*Δ strain derived from the W303 background to CFW when tested on glucose plates. We therefore repeated the assays using YEPDA supplemented with CFW or Congo red, i.e. with compounds that affect the yeast cell wall. Figure 6 shows that BY-p_TEF_-*UTH1* was significantly more sensitive to CFW and Congo red than the parental strain when grown on YEPDA, while we did not observe any significant differences in the sensitivity of BY-*uth1*Δ. However, the resistance of BY-*uth1*Δ and BY-p_TEF_-*UTH1* to boric acid on YEPDA plates was similar to that observed on GMA plates, only the effect of boric acid treatment was milder on YEPDA. Sensitivity of BY-*uth1*Δ strain to boric acid was significantly decreased on YEPDA-erg plates under hypoxic/anoxic conditions (Figure 7A) where the Uth1p-HA level is decreased (Figure 4A). As expected, changes in oxygen tension did not affect sensitivity of BY-p_TEF_-*UTH1* strain (where native control of *UTH1* gene expression is abolished) to CFM and Congo Red (Figure 7B and not shown). As shown in Figures 1 and 2, the profiles of SUN protein production and secretion in relation to distinct growth phases of yeast population are similar. The observed differences in the cell sensitivity of SUN-gene-deleted strains on YEPDA and GM seem therefore not simply related to the level of SUN protein production on the two media.

## Discussion

The SUN family is comprised of a group of fungus-specific proteins exhibiting high similarity, especially in their C-terminal domain (Table 1) [13], [16]. SUN family members have been predicted to be involved in various unrelated cellular processes, such as mitochondrial biogenesis and autophagy (mitophagy), cytokinesis, cell wall structure and DNA replication [7], [8], [17]. In addition, the β-glycosidase activity of these proteins was predicted on the basis of the homology of SUN proteins to the β-glycosidase of *C. wickerhamii* [14]. Localization of the three SUN proteins of *S. cerevisiae* Sun4p, Uth1p and Sim1p to the cell wall was shown [6], together with a secondary localization of Uth1p and Sun4p to the mitochondria.

In this paper we show that three of the SUN family proteins, Uth1p, Sun4p and Sim1p, are released from cells to the extracellular space during cultivation in both liquid cell cultures and in colonies growing on solid media. As for the fourth member of the group, Nca3p, its extracellular localization was not detected and even the cellular level of this protein was below the detection limit in colonies. In exponentially growing liquid cultures, all SUN proteins are present in high amounts in the cellular fractions, while their cellular concentrations decrease later when the cultures enter the postdiauxic and stationary phase. Even exponentially growing cells are able to release a detectable amount of Sun4p and Sim1p; this ability however gradually increases at the end of the exponential growth phase, especially with Sim1p. This, together with an increasing amount of cell biomass, leads to an accumulation of high concentrations of both proteins in the extracellular fluid. In contrast, the extracellular amount of Uth1p released by a biomass unit correlates with its intracellular concentration, which indicates that a fraction of Uth1p is always released when this protein is produced. In addition, it seems that at least extracellular Uth1p is partially degraded, as its absolute amount in the medium decreases in the stationary phase when the cells stop releasing it. Due to their continuous release from the cells, we cannot assess the extracellular stability of the other two proteins, Sun4p and Sim1p. In colonies, the profile of the extracellular level of Uth1p and Sun4p roughly corresponds to the cellular concentration of these proteins. As in liquid cultivations, the intracellular concentration of Sim1p drops rapidly even in relatively young 5-days-old colonies and the protein is released into the extracellular space over the next 15 days, which means that its concentration relative to a unit of biomass is maintained at a constant level. Interestingly, while the molecular weight (Mw) of extracellular Sim1p and Uth1p corresponded to that of the cellular proteins, the extracellular Sun4p variant is about 20 kDa shorter than the intracellular one, which indicates either Sun4p processing during its release or a different level of modification (possibly glycosylation). In addition, a smaller amount of Sun4p protein with an even higher Mw than the Mw of intracellular Sun4p was present in the extracellular space. This Mw difference of cellular versus extracellular Sun4p was not detectable in liquid cultivations in respiratory GM medium, while both forms (98 kDal and 77 kDal) were identified in fermentative YED medium. The Mw of intracellular Uth1p (62 kDa), Sun4p (98 kDa) and Sim1p (122 kDa) roughly corresponded to the Mws determined previously [6]. The differences from the Mws calculated from gene sequences (Table 1) were attributed to a high level of glycosylation of SUN proteins [6]. The Mw of Nca3p (57 kDa) that we observed was also higher than the Mw predicted from the sequence (35.4 kDa), suggesting that this protein is also modified. According to the prediction (Table 1), all four proteins contain multiple O-glycosylation sites and, in addition, Sun4p and Nca3p each contain one N-glycosylation site.

**Figure 8:**
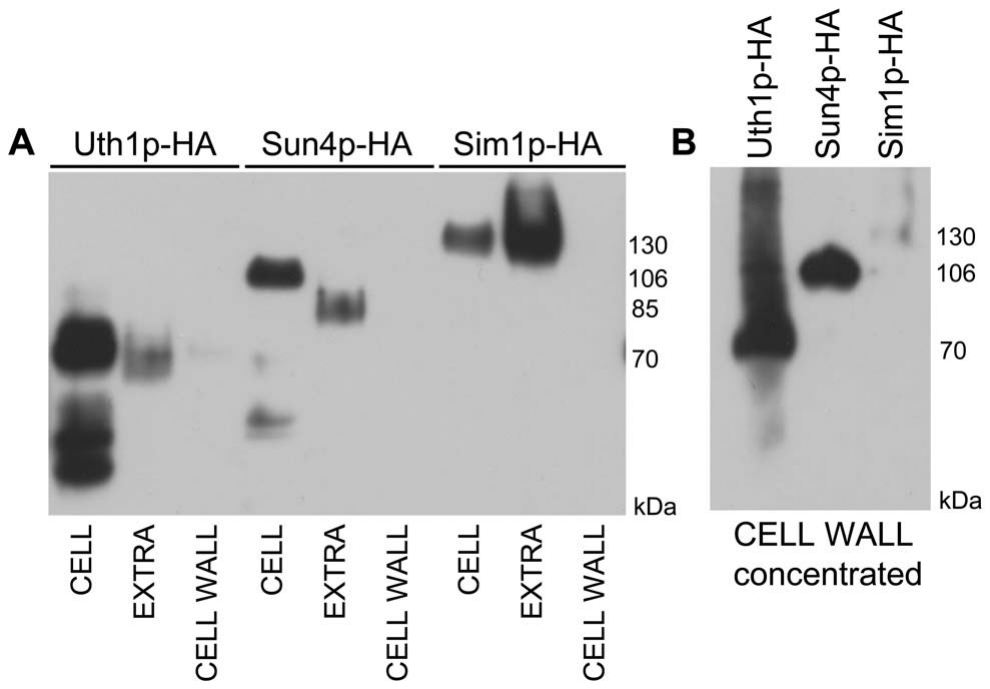
Localization of SUN proteins in cells from 3-days-old colonies grown on GMA plates. **A**, Protein amounts in cell lysates (CELL), extracellular extracts (EXTRA) and the purified cell walls (CELL WALL), respectively, prepared from colonies of BY-Uth1p-HA, BY-Sun4p-HA and BY-Sim1p-HA strains. The standardized amounts of lysate-proteins were loaded onto the gel and corresponding amounts of extracellular extracts and cell wall extracts. **B,** Proteins from 20-times concentrated cell wall extracts used in panel A.

What could be the reason for the release of the three SUN proteins from cells? Several proteins attached to the cell wall have been described to be also present in free form in the extracellular space, including the surface adhesin Flo11p [24]. The functions of these released protein variants are mostly unknown. One can speculate that SUN proteins may participate in remodeling the cell wall during the exponential-to-diauxic/stationary phase transition and in stationary cells they could affect cell wall structures from the “outside”. SUN proteins may affect cell morphogenesis, as was described for *C. albicans* Sun41p, which is involved in hyphae formation [15], [16], [25] and possibly secreted [25]. A second possibility that cannot be excluded is that these proteins are removed from the cell wall after accomplishing their task during cell division and septation, and that their presence in the extracellular medium is just a consequence of this release. However, comparison of the level of cellular, extracellular and cell-wall attached Uth1p-HA, Sun4p-HA and Sim1p-HA proteins from 3-days-old GMA grown colonies showed much smaller amount of Uth1p-HA and Sun4p-HA and almost no Sim1p-HA in purified cell wall fractions when compared to cellular and extracellular fractions (Figure 8). In addition, presence of these proteins also affects the sensitivity of aged colony cells to zymolyase (Figure S2) and in particular Sim1p accumulates in the extracellular space of ageing colonies as well as in liquid cultures and is therefore produced and directly released by stationary cells. All these findings favor the first possibility.

An increased resistance of the strain with a deleted *UTH1* gene to zymolyase treatment has been reported [8]. Here we show that the deletion of any of the four SUN genes confers cells grown on GMA plates with high resistance to this lytic enzyme. As the increased resistance to zymolyase was attributed to an increased level of β-1-6 glucan in the yeast cell wall [26], we can speculate that the deletion of any of the SUN genes leads to a remodeling of the yeast cell wall accompanied by an increasing in β-1-6 glucans. The finding that the level of β-glucans increased in the *uth1*Δ strain [8] supports this. Interestingly, an increased resistance of the SUN-gene-deleted strains to zymolyase was not only apparent in cells with a high cellular concentration of SUN proteins, but also later when SUN proteins are released from the cells. For example, in 20-days-old colonies, most of the Sim1p is released from the cells, but deletion of the *SIM1* gene still increases cell resistance to zymolyase (Figure S2). These data indicate that although expression of *UTH1* and *SIM1* decreases during colony ageing [27], [28] and the expression of all three genes *SUN4*, *UTH1* and *SIM1* is decreased during the entry of liquid culture cells to the stationary phase [29], which both corresponds to an observed decrease in SUN protein cellular concentration, these proteins, and especially Sim1p, can persist much longer outside the cells and could play important roles, even in later phases of cell culture development.

In addition to the increased resistance to zymolyase observed in strains with any of their SUN genes knocked out, the BY-*uth1*Δ strain grown on GMA plates was more sensitive than the parental BY4742 strain to other compounds affecting the composition and/or structure of the yeast cell wall. Three of these compounds, Calcofluor white, Congo red and boric acid have been described to be linked to an increased cellular chitin content in treated cells [30]–[32]. The increased sensitivity of the BY-*uth1*Δ strain to these compounds together with the previously observed decrease in chitin content [8] suggests that Uth1p could be somehow involved in the synthesis or assembly or deposition or correct localization of cellular chitin. In addition, both the BY-*uth1*Δ and BY-*sim1*Δ strains were more sensitive to a low concentration of SDS detergent than the parental BY4742, BY-*sun4*Δ and BY-*nca3*Δ strains. The fact that SDS affects cell wall integrity [33] and observed sensitivity to this compound suggests that the cell walls of the BY-*uth1*Δ and BY-*sim1*Δ strains are more fragile than those of the cells of the other two strains, although all SUN gene delta strains exhibit an increased resistance to zymolyase treatment. SDS apparently affects different components of yeast cell surface structures than zymolyase does. The BY-*uth1*Δ sensitive phenotype to all four compounds can be completely reversed by *UTH1* gene overexpression controlled by the constitutive strong TEF promoter. The BY-p_TEF_-*UTH1* strain even reaches a resistance to boric acid and SDS that surpasses the resistance of the parental strain. The finding that the presence of Uth1p is important for yeast resistance to CFW and Congo red contradicts the previous observation of Ritch et al [8] that a *uth1*Δ strain derived from a different background (W303) is more resistant to these compounds according to drop tests. As this difference could be due to either the different strain backgrounds or the different growth conditions used in the screen, we tested BY-*uth1*Δ strain sensitivity using YEPDA plates, as were used by Ritch et al [8]. The results clearly showed that BY-*uth1*Δ strain sensitivity to CFW and Congo red is affected by the carbon source, being higher on respiratory GMA plates and the same on fermentative YEPDA plates (where Uth1p overproduction leads to increased sensitivity to these compounds) as compared with the sensitivity of the BY4742 parental strain. On the other hand, BY-*uth1*Δ was more sensitive to boric acid than the parental strain on both media. Thus, Uth1p could have a different effect on yeast cell walls under different environmental conditions. It was shown that different carbon sources significantly affect the chitin and β-glucan composition of the cell wall [26]. One can therefore speculate that Uth1p (and maybe also other SUN proteins) could be involved in such an environmental adaptation.

*SUN41* of *C. albicans* is one of the genes induced by hypoxia [34]. Our data showed that in *S. cerevisiae*, the production of Uth1p and Sim1p is decreased under conditions of decreased oxygen (anoxia or 1% oxygen), while the production of Sun4p was relatively stable, exhibiting no dependence on the oxygen level. These findings imply that in addition to group of cell wall DAN/TIR mannoproteins that is comprised of members regulated by oxygen in various ways [35], the SUN-family proteins represent another group of proteins that may be involved in cell wall remodeling at various oxygen concentrations. This prediction is supported by the finding that sensitivity of BY-*uth1*Δ strain to boric acid was diminished under conditions of decreased oxygen tension, i.e. under conditions where cells decrease Uth1p-HA protein production and thus probably do not need this protein.

In summary, our new data suggest that SUN-family proteins are regulated differently during particular phases of yeast culture development and under different environmental conditions, apparently being involved in remodeling of the cell wall and changes in its resistance to extracellular compounds. In addition, the finding that cell sensitivity to boric acid, i.e. to a fungistatic compound that is used in the medical treatment of recurrent and resistant yeast vaginitis, is dependent on the presence (and level) of Uth1p makes this protein an interesting target for studies of boric acid’s action.

## Materials and Methods

### Yeast Strains and Media

The strains used in this study (Table 2) were derived from *Saccharomyces cerevisiae* BY4742 (MATα, *his3Δ1*, *leu2Δ0*, *lys2Δ0*, *ura3Δ0*) obtained from the EUROSCARF collection. Cells were grown at 28°C either in liquid YED medium (1% yeast extract, 2% glucose), or in liquid GM medium (1% yeast extract, 3% glycerol, pH 5) or on GMA agar (GM, 2% agar, 30 mM CaCl_2_, pH 5). For spot assays, GMA agar was supplemented with 0.012% SDS, 0.4% boric acid, 800 µg/ml Congo red and 1 mg/ml Calcofluor white (CFW). YEPDA agar (YED, 1% pepton, 2% agar,) was supplemented with 0.2% boric acid, 800 µg/ml Congo red and 600 µg/ml Calcofluor white. YEPDA-erg, YEPDA supplemented with 0.5 ml/100 ml ergosterol solution (2 mg ergosterol, 800 µl ethanol, 200 µl Tween 80) was used for experiments under anoxic conditions. SDA agar (2% glucose, 100 mM KH_2_PO_4_, 15 mM (NH_4_)_2_SO_4_, 0.8 mM MgSO_4_, 0.15% Wickerham’s yeast nitrogen base supplemented with 150 µg/ml of uracil, leucine and lysine, 2% agar) and YEPDA-G418 agar (YEPDA with 400 µg/ml geneticin G418) were used for the selection of transformants.

### Strain Construction

A hemagglutinin protein (HA) gene tag and artificial constitutive TEF promoter (p_TEF_) were fused to the appropriate gene directly in the chromosome [36]. For amplification of the cassette, we used the primers specific for the *UTH1*, *NCA3*, *SUN4* and *SIM1* genes and the appropriate plasmid as the template. Strains were prepared using the plasmids pYM15 (HA-tag) and pYM-N18 (p_TEF_) [37]. The cassettes were amplified using specific primers (Table S1) and transformed according to [38] into BY4742 cells. Transformants were selected either on SDA agar with auxotrophic supplements or on YEPDA-G418. Correct genomic integration of the cassette was verified by PCR using specific primers and by sequencing.

### Extracellular and Cellular Protein Extraction and Separation using SDS–PAGE

Cell lysates and extracellular material from colonies was collected as follows: The biomass of whole colonies was collected, weighed and washed with an equal volume of the 10 mM MES buffer, pH 6 supplemented with protease inhibitors (Complete, EDTA-free protease inhibitor mixture (Roche Applied Science), 100 mM PMSF (Phenylmethylsulfonyl fluoride, Sigma) in isopropanol and 1 mM AEBSF (4-(2- aminoethyl) benzenesulfonyl uoride, Sigma)), i.e. for example 10 µg of the biomass was washed with 10 µl of the buffer. After the centrifugation (5 min, 4°C, 1180 g) supernatants and sediments with cells were collected separately and stored at −75°C. Supernatants were loaded onto the slots of SDS-PAGE gel in equal volumes which were equivalent to the amount of washed cells.

Cell lysates and extracellular proteins from liquid cultures were prepared as follows: The same cell biomass was collected by the centrifugation (5 min, 4°C, 1180 g) of a specific volume of cell culture (calculated according to the OD_600_ of the culture) grown in liquid GM/YED medium. The collected cells were used for the preparation of cell lysates containing SUN^cell^ proteins. SUN^ex^ proteins from the medium supernatant were precipitated with 100% Trichloroacetic acid added in a 1∶10 ratio and kept on ice for 2 hours for quantitative precipitation. Precipitated proteins were centrifuged (15 min, 4°C, 12000 g), washed twice with acetone and resuspended in 200 µl of 10 mM MES buffer, pH6 with protease inhibitors. The amounts of extracellular proteins were loaded onto the slots of SDS-PAGE to be equivalent to the amount of cells that produced those proteins as well as cellular proteins loaded onto the gel.

The total-cell lysates were prepared from cells and collected as indicated above. All steps were performed at 4°C. The cells were broken with glass beads in 10 mM MES buffer, pH 6 with protease inhibitors in a FastPrep (Qbiogene). Cell debris was removed by centrifugation at 1000 g for 3 min and subsequently 2000 g for 5 min. The protein concentration in the supernatant was determined using a protein detection kit (Bio-Rad) and the aliquots were stored at −75°C.

### Cell Wall Isolation

Cells were disrupted as in the case of cell lysate preparation. Sediments obtained after 1000 g and 2000 g centrifugations were combined, cell walls isolated as described [39], washed with ice-cold 10 mM Tris-HCl, pH 7,4, 1 mM PMSF (Phenylmethylsulfonyl fluoride, Sigma) and centrifuged at 1000 g, 4°C for 10 min. Sediment was washed with 50 ml ice-cold wash solution A (1 mM PMSF), and subsequently with wash solution B (5% NaCl, 1 mM PMSF), C (2% NaCl, 1 mM PMSF) and D (1% NaCl, 1 mM PMSF); each of the washings was repeated four times. Isolated cell walls were suspended in 100 µl of 10 mM MES buffer, pH 6 supplemented with protease inhibitors. Cell wall proteins were solubilized and denatured by Laemmli sample buffer and subjected to SDS-PAGE.

### Determination of Amount of Particular SUN Proteins

The proteins of cell lysates were denatured in Laemmli sample buffer and separated by SDS-PAGE using 9% gels. 20 µg of protein was loaded onto the slots with the exception of Nca3p-HA, where 30 µg was loaded. After transfer to a PVDF membrane (Immobilon-P, Millipore), the amount of loaded proteins was verified by Coomassie blue staining of each membrane (loading control). The HA-tagged SUN family proteins were detected by mouse anti-HA antibodies (Cell Signaling Technology) in combination with goat anti-mouse IgG-HRP as the secondary antibody (Santa Cruz Biotechnology). The peroxidase signal was visualized with Super Signal West Pico (Pierce) on Super RX medical X-ray film (Fuji). The level of the individual protein was evaluated by UltraQuant 6.0. To minimize an effect of band saturation, less exposed Western blots were usually used for the quantification. Only in those cases where large differences in signal intensity were present among quantified samples, the quantification of most concentrated sample/s could be partially affected by their saturation.

### Cell Wall Sensitivity to Zymolyase

The cell biomass of whole colonies grown on GMA plates was collected and resuspended in 20 mM potassium phosphate buffer, pH7.4 to a cell concentration providing OD_600_ = 0.4 after 100-fold dilution. After 1 min of preheating, Zymolyase 100 T (Zymo Research) was added (time zero) to a final concentration of 0.2 U/µl and incubated at 30°C for 20 min. Cell wall resistance to Zymolyase was determined as the decrease in OD_600_ of a cell suspension; i.e. in 2 min intervals, 10 µl of treated cell suspension was diluted 100 times into 1 ml of distilled water and the OD_600_ was determined.

### Spot Assays

To determine sensitivity of cells to toxic compounds, particular strains were grown overnight on YEPDA agar at 28°C. To compare the viability of colony population of different strains, the giant colonies were grown 6 per plate on GMA at 28°C [28] and the whole population of a colony was harvested.

For the spot tests an equal wet cell biomass of each strain was diluted in distilled water as follows: A series of 10-fold dilutions were prepared in water over a range of concentrations from 10^−1^ to 10^−5^ relative to the initial culture. Spots of 5 µl from each dilution series were then plated on the indicated media and cultivated at either 28 or 37°C for 4 or 7 days, depending on the particular medium and treatment. All spot assays were repeated at least three times and a representative experiment is shown.

### Cell Incubation in Anoxomat Device

Cells were incubated under either hypoxic (1% O_2_, 79.3% N_2_, 10.3% CO_2_, 9.5 H_2_) or anoxic (0% O_2_, 80% N_2_, 9.9% CO_2_, 9.9 H_2_) conditions. For incubation, we used an Anoxomat™ device (Mart Microbiology b.v.).

## Supporting Information

Figure S1 **The viability of BY4742, BY-** ***uth1*** **Δ, BY-** ***sun4*** **Δ, BY-** ***sim1*** **Δ and BY-** ***nca3*** **Δ populations during 30 days of colony development.** At specified time points the whole colony populations were harvested and viability of particular strains was compared by spot assays on YEPDA plates.
(PDF)

Figure S2 **Resistance of cells from 20 days old colonies grown on GMA to zymolyase presented as decrease in density of cell suspension.** Values represent averages from 4 independent biological replicates for the mutant strains and 6 replicates for the wt; the significance of the difference between BY4742 and the other four strains was determined using two-way ANOVA with p<0.05.
(PDF)

Figure S3 **Loading controls for Western blots shown in** **Figures 1** **,** **2** **,** **3** **and** **4** **.**
(PDF)

Figure S4 **Sensitivity of strains deficient in SUN proteins to toxic compounds either on respiratory GMA (A) or on fermentative YEPDA (B) agar.** Quantification of drop assays shown at Figures 5 and 6.
(PDF)

Table S1 **List of the primers.**
(PDF)
