# Supplementary material for: SUN Family Proteins Sun4p, Uth1p and Sim1p Are Secreted from Saccharomyces cerevisiae and Produced Dependently on Oxygen Level
Source: PLoS One. 2013 Sep 11;8(9):e73882. doi: 10.1371/journal.pone.0073882 (PMC3770667; doi:10.1371/journal.pone.0073882)
Supplement: Figure S1 — The viability of BY4742, BY- uth1 Δ, BY- sun4 Δ, BY- sim1 Δ and BY- nca3 Δ populations during 30 days of colony development. At specified time points the whole colony populations were harvested and viability of particular strains was compared by spot assays on YEPDA plates. (PDF) [file pone.0073882.s001.pdf]

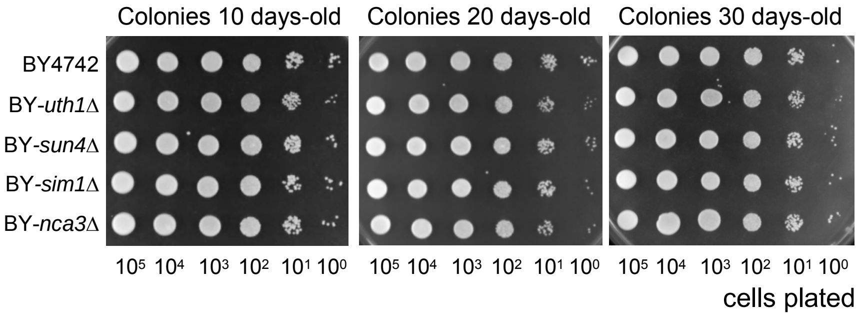

Figure S1

Viability of BY4742, BY-*uth1*Δ, BY-*sun4*Δ, BY-*sim1*Δ and BY-*nca3*Δ strains within colonies grown on GMA during 30 days of colony development. At specified time points the whole colony populations were harvested and viability of particular strains was compared by spot assays on YEPDA plates.
