# Supplementary material for: SUN Family Proteins Sun4p, Uth1p and Sim1p Are Secreted from Saccharomyces cerevisiae and Produced Dependently on Oxygen Level
Source: PLoS One. 2013 Sep 11;8(9):e73882. doi: 10.1371/journal.pone.0073882 (PMC3770667; doi:10.1371/journal.pone.0073882)
Supplement: Figure S2 — Resistance of cells from 20 days old colonies grown on GMA to zymolyase presented as decrease in density of cell suspension. Values represent averages from 4 independent biological replicates for the mutant strains and 6 replicates for the wt; the significance of the difference between BY4742 and the other four strains was determined using two-way ANOVA with p<0.05. (PDF) [file pone.0073882.s002.pdf]

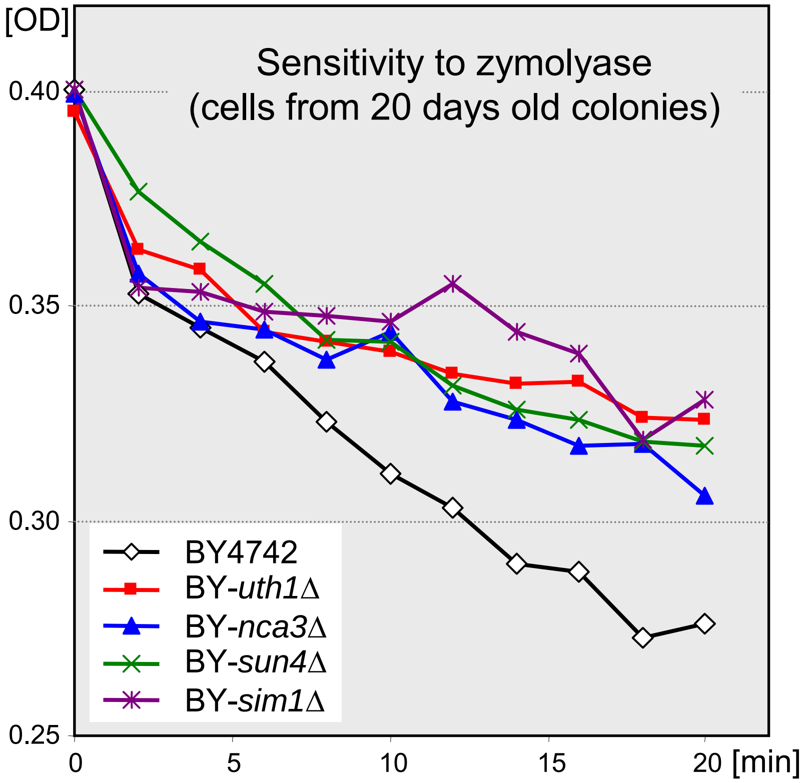

Figure S2

Resistance of cells from 20 days old colonies grown on GMA to zymolyase presented as decrease in density of cell suspension. Values represent averages from 4 independent biological replicates for the mutant strains and 6 replicates for the wt; the significance of the difference between BY4742 and the other four strains was determined using two-way ANOVA with $p < 0.05$.
