# Supplementary material for: SUN Family Proteins Sun4p, Uth1p and Sim1p Are Secreted from Saccharomyces cerevisiae and Produced Dependently on Oxygen Level
Source: PLoS One. 2013 Sep 11;8(9):e73882. doi: 10.1371/journal.pone.0073882 (PMC3770667; doi:10.1371/journal.pone.0073882)
Supplement: Figure S3 — Loading controls for Western blots shown in Figures 1 , 2 , 3 and 4 . (PDF) [file pone.0073882.s003.pdf]

A. Loading controls for Figure 1A

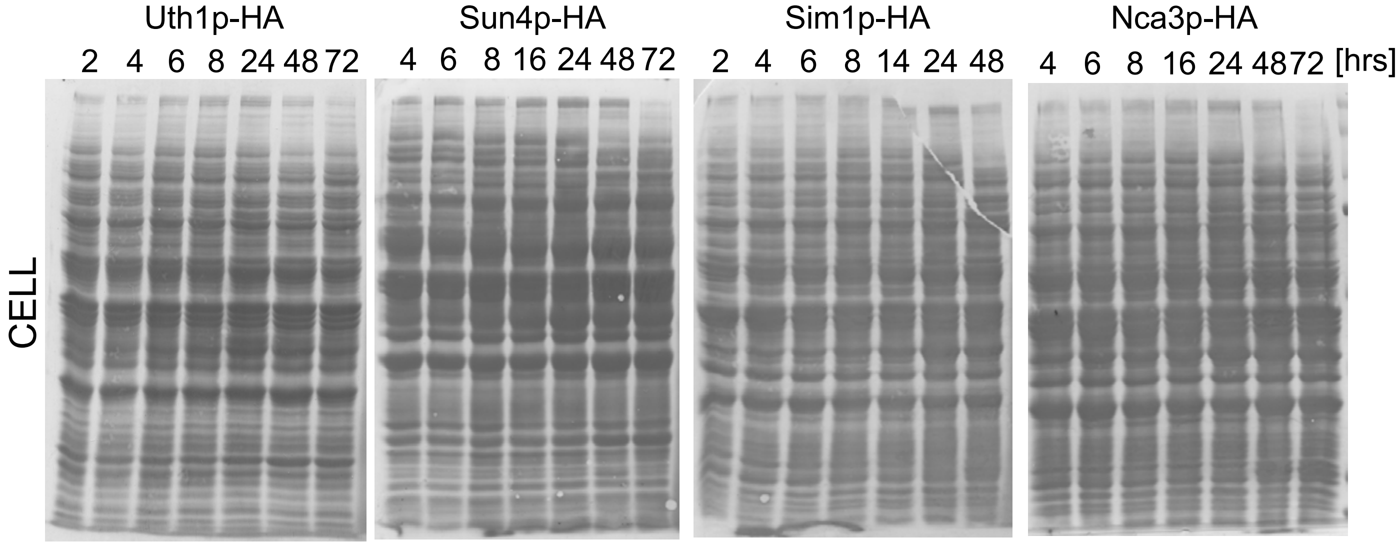

B. Loading controls for Figure 2

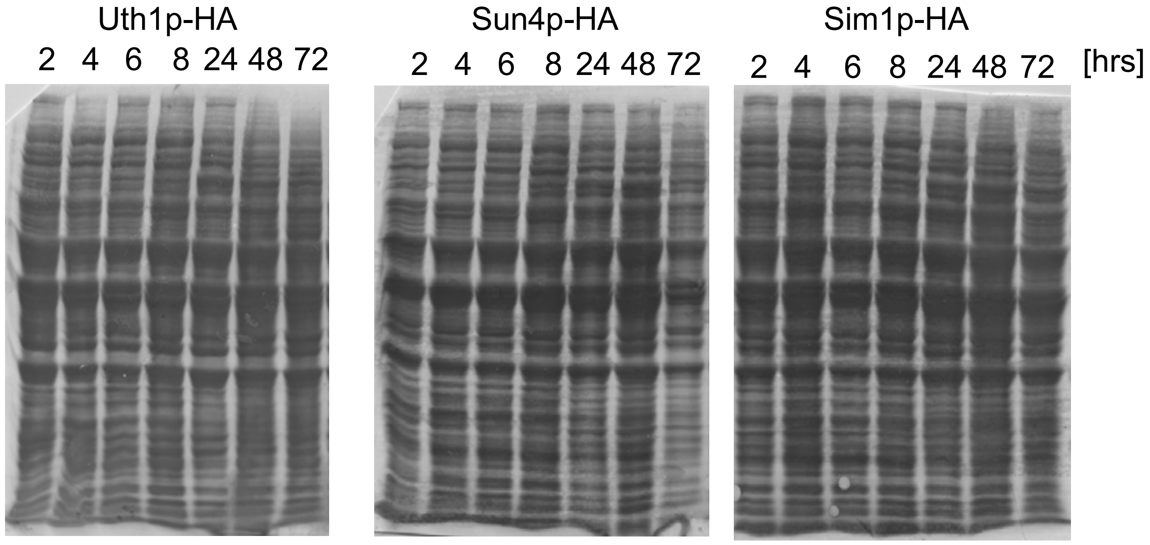

C. Loading controls for Figure 3

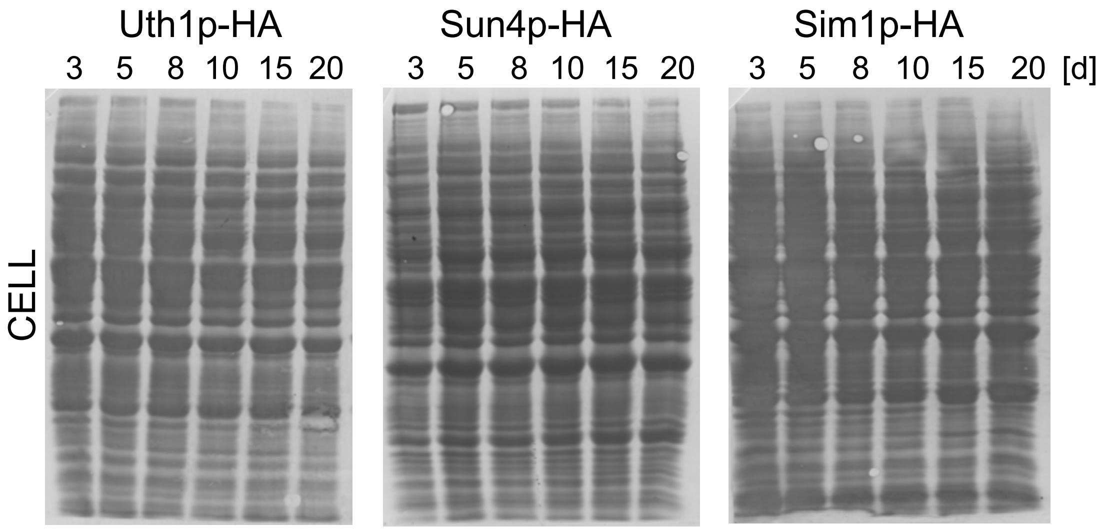

D. Loading controls for Figure 4

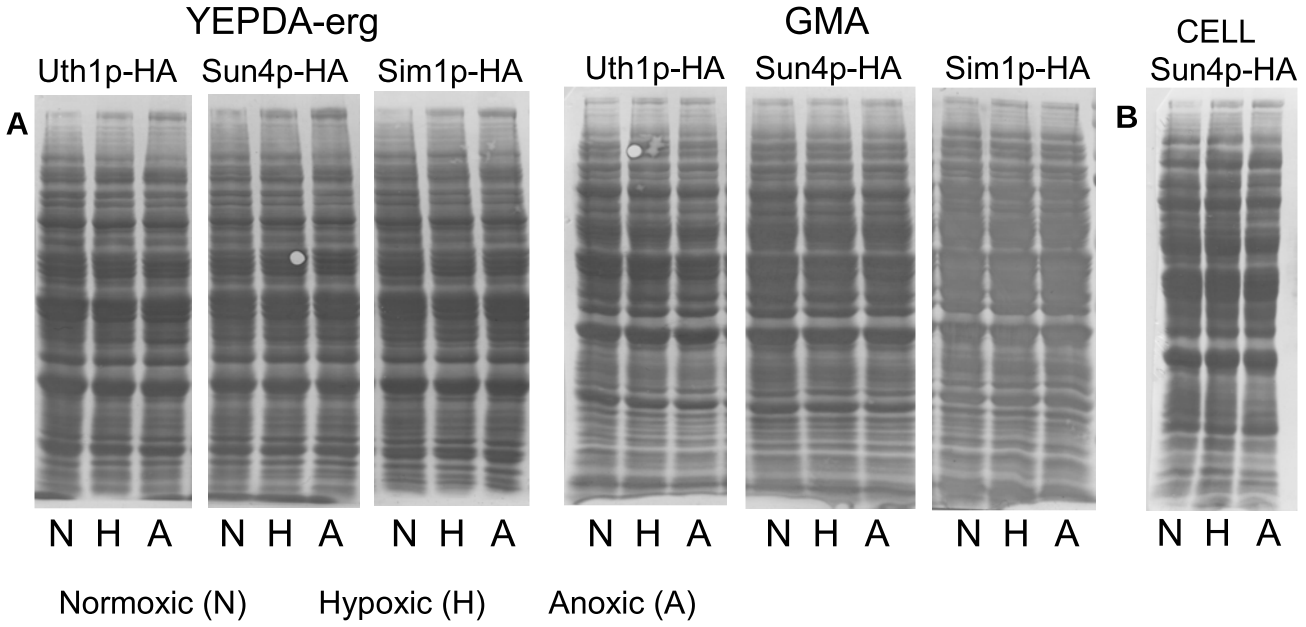

Figure S3: Loading controls for Western blots shown in Figures 1, 2, 3 and 4.
