# Supplementary material for: SUN Family Proteins Sun4p, Uth1p and Sim1p Are Secreted from Saccharomyces cerevisiae and Produced Dependently on Oxygen Level
Source: PLoS One. 2013 Sep 11;8(9):e73882. doi: 10.1371/journal.pone.0073882 (PMC3770667; doi:10.1371/journal.pone.0073882)
Supplement: Figure S4 — Sensitivity of strains deficient in SUN proteins to toxic compounds either on respiratory GMA (A) or on fermentative YEPDA (B) agar. Quantification of drop assays shown at Figures 5 and 6. (PDF) [file pone.0073882.s004.pdf]

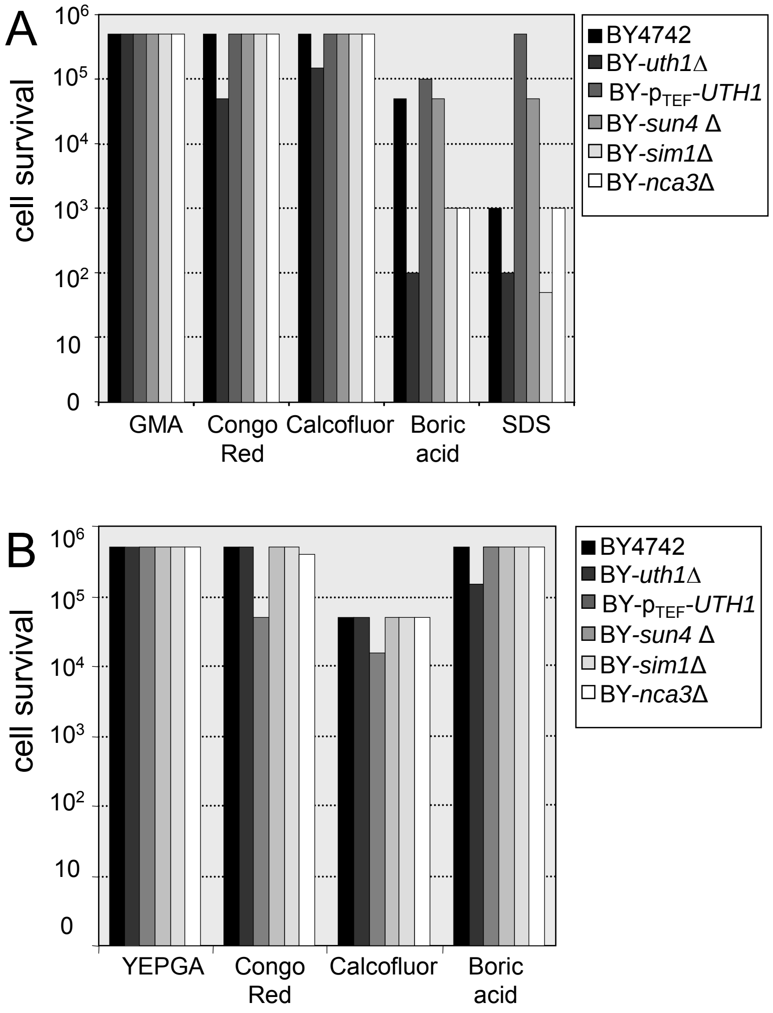

Figure S4

Sensitivity of strains deficient in SUN proteins to toxic compounds either on respiratory GMA (A) or on fermentative YEPDA (B) agar. Quantification of drop assays shown at Figures 5 and 6.
