# Supplementary material for: SUN Family Proteins Sun4p, Uth1p and Sim1p Are Secreted from Saccharomyces cerevisiae and Produced Dependently on Oxygen Level
Source: PLoS One. 2013 Sep 11;8(9):e73882. doi: 10.1371/journal.pone.0073882 (PMC3770667; doi:10.1371/journal.pone.0073882)
Supplement: Table S1 — List of the primers. (PDF) [file pone.0073882.s005.pdf]

Table S1: List of the primers

primer	sequence (5'→3')	purpose
UTH1-HA-fw	GAC GGT TGT ACT GTT TCA GTT ACT TCT GGT TCT GCT AAC TTT GTC TTC TAC CGT ACG CTG CAG GTC GAC	C-terminal fusion of *UTH1* with HA
UTH1-HA-rev	AAA AAA GTA CTA GCA AAA GCT TAT TTG CAA TAT TCA AGG AAA AAA GGC CTA ATC GAT GAA TTC GAG CTC G	C-terminal fusion of *UTH1* with HA
NCA3-HA-fw	ATG GTT GCA CAG TTT CTG TTT TAT CTG GAT CTG CTG AAT TTG TTT TCT ATC GTA CGC TGC AGG TCG AC	C-terminal fusion of *NCA3* with HA
NCA3-HA-rev	ATT GAG GGT ATC AAA ATA CAA GAC ATT CTT TTA CCG AAA AGA AGA ATG ACA TCG ATG AAT TCG AGC TCG	C-terminal fusion of *NCA3* with HA
SUN4-HA-fw	GTT GTA CCG TTT CCG TTA CTG CTG GCA AAG CTA AGT TTG TTC TAT ACA ACC GTA CGC TGC AGG TCG AC	C-terminal fusion of *SUN4* with HA
SUN4-HA-rev	GAA TGG GGT AAT AAT ACA ATC AAC TTA CTC AAC TGT TGA TGC GCC TAA GTA TCG ATG AAT TCG AGC TCG	C-terminal fusion of *SUN4* with HA
SIM1-HA-fw	GTG TAC CGT CTC TGT TAC TTC CGG TAA AGC TCA TTT CGT CTT ATA CAA TCG TAC GCT GCA GGT CGA C	C-terminal fusion of *SIM1* with HA
SIM1-HA-rev	GAT GTG TTC GAA AAA AGA AAA AAA AAA GGA AAA GTA GTA GTC ACG TAG CAT CGA TGA ATT CGA GCT CG	C-terminal fusion of *SIM1* with HA
p_{TEF}-UTH1-fw	CCT TCC TTT AAA CAA AAA TTT ACC CTC CCT TAA TTT TTC AAG AAA TTC CAG TAT GCG TAC GCT GCA GGT CGA C	Insertion of p_{TEF} in front of *UTH1*
p_{TEF}-UTH1-rev	CTG GAG CGG CCA AGA CGG CGG TGG AGG CTG ATA AAG CTA ATA GAG CGG ATA ATT TCA TCG ATG AAT TCT CTG TCG	Insertion of p_{TEF} in front of *UTH1*
